# Comparison of regression for blood ALP levels using methods of the Japan Society of Clinical Chemistry and the International Federation of Clinical Chemistry and Laboratory Medicine in bovine, canine, feline, and human testing

**DOI:** 10.1371/journal.pone.0253396

**Published:** 2021-06-16

**Authors:** Akihisa Hata, Noboru Fujitani, Masahiro Takeshita, Chie Tanaka, Noriko Matsuda, Michiko Takaishi, Takako Shimokawa Miyama, Fumio Hoshi

**Affiliations:** 1 Faculty of Veterinary Medicine, Okayama University of Science, Imabari, Ehime, Japan; 2 Biomedical Science Examination and Research Center, Okayama University of Science, Imabari, Ehime, Japan; 3 Ehime Rinken Inc., Matsuyama, Ehime, Japan; University of Lincoln, UNITED KINGDOM

## Abstract

Livestock and companion animal health have a direct impact on human health. Research on clinical laboratory technology for veterinary medicine is as important as that on human laboratory technology. Reagents and analysis equipment for human medical laboratory tests are often used in veterinary medicine. Medical laboratories in Japan utilize the Japan Society of Clinical Chemistry (JSCC) method for blood alkaline phosphatase (ALP) analysis. The International Federation of Clinical Chemistry and Laboratory Medicine (IFCC) method is used worldwide for ALP catalytic concentration measurement. When the IFCC method is used, human blood ALP activity is approximately one-third of the JSCC method’s activity. The JSCC method for ALP measurement was switched to the IFCC method in medical laboratories in Japan in April 2020 for global standardization purpose. It is uncertain whether conventional JSCC method reagents will continue to be supplied. In veterinary medicine, the relationship between the JSCC and IFCC methods in terms of ALP measurement is almost unclear. This study investigated the regression between JSCC and IFCC methods measuring ALP in bovine, canine, feline, and human. The regression formulas for bovine, canine, feline, and human ALP values using the conventional JSCC (*x*) and IFCC (*y*) methods are *y* = 0.379*x* + 0.124, *y* = 0.289*x* + 8.291, *y* = 0.358*x* + 0.432, and *y* = 0.337*x* + 2.959, respectively. These results suggested that the IFCC method measurement could be estimated by approximately one-third of the JSCC method measurement in animal species such as bovine, canine, and feline. By applying the conversion factors proposed in this study, a very good correlation could be obtained between the two methods for each animal.

## Introduction

Adequate quality control and accurate testing in veterinary medicine are necessary for proper care of livestock and companion animals. Animal health has a direct impact on human health. Research on clinical laboratory technology for veterinary medicine is as important as human laboratory technology. Reagents and analysis equipment for human medical laboratory tests are often used in veterinary medicine. Therefore, veterinary laboratory tests are affected by any disruption in the supply of human clinical test reagents or changes in reagent composition.

Alkaline phosphatase (ALP) concentration is known to increase in primary liver disease, drug-induced liver injury, pancreatitis, hypercortisolism, and metabolic bone disease [1]. Medical laboratories in Japan utilize the Japan Society of Clinical Chemistry (JSCC) method for blood ALP catalytic concentration measurement. However, the International Federation of Clinical Chemistry and Laboratory Medicine (IFCC) method is used worldwide for ALP catalytic concentration measurement. For global standardization, the JSCC method of ALP was switched to the IFCC method in medical laboratories in Japan in April 2020. It is uncertain whether conventional JSCC method reagents will continue to be supplied. The JSCC and IFCC methods differ in terms of the type of buffer solution used. While 2-ethylaminoethanol buffer is used in the JSCC method [2], 2-amino-2-methyl-1-propanol buffer is utilized in the IFCC method [3]. This difference results in the IFCC method yielding human blood ALP estimates at levels of approximately one-third more than those of the JSCC method [4]. In a previous study, we have clarified the relationship between the JSCC and IFCC methods in terms of ALP measurement in canine species [5], although the relationship is almost unclear in most other animals. The measured values of the JSCC and IFCC methods cannot be used interchangeably in most animal species. This primary aim of this study was to clarify the relationship between the JSCC and IFCC methods for felines, which have the second-highest number of patients in companion animals after canines, and in bovines, which have the highest number of patients as industrial animals. Further, we analyzed human and canine ALP specimens and compared the relationships between JSCC and IFCC methods in four animal species. If the difference in these regression values between animal species is small, it can be inferred that the same tendency may exist in other animal species. This animal species comparison was the second purpose of this study.

We collected blood specimens from adult bovines, canines, and felines as well as humans. The regression between ALP values determined using the JSCC and IFCC methods was analyzed in these blood specimens. The regression formulas obtained in each animal species using these data were compared to confirm animal species differences. When the JSCC and IFCC values co-exist, these regression formulas could help avoid confusion in companion animal and industrial animal medicine.

## Materials and methods

### Ethical approval

In the present study, residual blood from human and animal biochemical tests was used. Animals were not subject to ethical considerations at the Okayama University of Science ethics committee on clinical investigation (examination number 2020–0007) because blood was not sampled from animals in this study. Adult human specimens were provided with full anonymity. The study plan was approved by the Ethical Committee for Medical and Health Research Involving Human Subjects of Okayama University of Science (approval number 2–11). The ethics committee determined that there was no need for consent to be obtained from the sample donor.

### Sample and data collection

Human specimens were completely anonymized, and measurements were completely separated from personal information. Individual information on bovines, canines, and felines was handled in the same manner.

A total of 116 bovines, 108 canines, and 103 feline residual blood samples from blood biochemistry analyses at the Biomedical Science Examination and Research Center, Okayama University of Science (Imabari, Ehime, Japan) were collected. In total, 100 human residual blood samples from blood biochemistry analyses at the Ehime Rinken Incorporated (Matsuyama, Ehime, Japan) were collected. We did not analyze multiple specimens from the same subject.

Blood samples did not include specimens of animals <1 year of age, and samples from humans did not include specimens of individuals under 20 years of age. Bovine samples included dairy (Holstein Friesian cattle, *N* = 105) and beef cattle (Japanese black cattle, *N* = 11). Pregnant animals were excluded.

Lithium heparin was used as an anticoagulant for canine and feline blood. No anticoagulant was used for bovine and human blood samples. Blood samples were centrifuged (1,500 × *g*, 10 min), after which sera or plasma was separated; they were frozen at −30°C until further analysis.

### Analysis of blood ALP activity

The blood ALP analysis was performed in the same manner as that mentioned in our previous study [5]. Commercially available JSCC transferable reagent kits were purchased from Fujifilm Wako Pure Chemical (Osaka, Japan) unless otherwise specified. Table 1 shows the ALP analysis reagents and measurement conditions in this study. These reagents are widely used in routine analysis methods in clinical laboratories in Japan. The enzyme calibrator Wako was used for calibration. Control Wako-I and Wako-II were used for quality control. The calibrator and control used were produced under the standard material producer certification (ISO 17034). The ALP value of the enzyme calibrator has been evaluated by employing the in-house standard measurement operation method of Fujifilm Wako Pure Chemical using the Japanese Committee for Clinical Laboratory Standards-certified standard material (JCCLS CRM-001). The ALP values of the Control Wako-I and -II have been evaluated by the JSCC and IFCC transferable methods using the enzyme calibrator. We used the Hitachi 3100 clinical analyzer (Hitachi High-Technologies Corp., Tokyo, Japan) for ALP analysis, wherein the reaction parameters for ALP analysis were set to the values recommended by the manufacturer. The control Wako-I and II values indicated by the manufacturer (standard deviation), analyzed by the JSCC method, were 206 (5.3) and 599 (12.7) U/L, respectively, while those analyzed by the IFCC method were 76 (2.1) and 250 (7.0) U/L, respectively. In our laboratory, as analyzed by the JSCC method, the assigned ALP values of Control Wako-I and -II were 203 and 600 U/L, respectively. As analyzed by the IFCC method, the assigned ALP values of Control Wako-I and -II were 74 and 249 U/L, respectively. For both JSCC and IFCC methods, the inter-assay coefficients of variation (CVs) using Control Wako-II were 0.82% and 0.68%, respectively. The intra-assay CVs using Control Wako-II were 0.79% and 0.74%, respectively.

**Table 1:** ALP analytical reagents and measurement conditions in this study.

Reagent		
Name of analytical method	JSCC transferable method	IFCC transferable method
Product name	ALP II-J2	ALP IFCC
Manufacturer	Fujifilm Wako Pure Chemical	
Contents of reaction (buffer) solution	2-Ethylaminoethanol	2-Amino-2-methyl-1-propanol
Contents of starting reagent (substrate) solution	4-Nitrophenyl phosphate, disodium salt, hexahydrate	
Lower limits of quantitation (U/L)	1.5	1
Upper limits of quantitation (U/L)	2000	700
Measurement condition		
Temperature (°C)	37	
Main wavelength (nm)	405	
Sub-wavelength (nm)	505	
Incubation time (min)	5	
Delay time (min)	1	1.5
Measurement interval (min)	4	3.5
Sample volume (μl)	3	4
Volume of reaction solution (μl)	200	160
Volume of starting reagent solution (μl)	50	40

### Statistical analysis

The statistical analysis was performed in the same manner as that mentioned in our previous study, as described below [5]. ALP values obtained using both the methods were plotted using a scatter diagram, wherein the x-axis represented the value obtained using the JSCC method and the y-axis represented that obtained using the IFCC method. Regression formulas were developed using standard major axis regression in Validation-Support/Excel Ver.3.5 (JSCC, Quality Management Expert Committee). The 95% confidence interval was calculated via bootstrapping.

We calculated each regression formula’s residual using the following formula: (actual measurement value using the IFCC method) − (IFCC value estimated using regression formula). Furthermore, we calculated each regression formula’s standardized residuals using the following formula: (residual) / (residual standard deviation).

We used the slope of the regression formulas of standard major axis regression analysis method as the conversion factors in this study.

## Results

### Blood ALP activity in each animal species

The range of ALP activity analyzed by the JSCC and IFCC methods was as follows; bovine, 28–259 and 10–98 U/L; canine, 15–1615 and 5–555 U/L; feline 15–534 and 5–181 U/L; and human, 89–388 and 33–143 U/L. Table 2 shows the median values and interquartile ranges of animal and human samples by both measurement methods. The range of ALP distribution for canines is wider than for other animal species.

**Table 2:** Regression analyses between the JSCC and IFCC methods in bovine, canine, feline, and human blood samples.

		Median (interquartile range) ALP activity		Regression formula (JSCC: ***x***, IFCC: ***y***)	***r***	95% confidence interval	
Species	***N***	JSCC (U/L)	IFCC (U/L)			Lower limit	Upper limit
**Bovine**	116	89 (63–133)	34 (24–51)	*y* = 0.379*x* + 0.124	0.996	*y* = 0.371*x* − 0.688	*y* = 0.389*x* + 0.859
**Canine** All	108	241 (139–536)	82 (46–168)	*y* = 0.289*x* + 8.291	0.990	*y* = 0.273*x* + 3.273	*y* = 0.308*x* + 12.738
Q1–3	81	196 (127–275)	64 (41–89)	*y* = 0.331*x* − 0.045	0.994	*y* = 0.321*x*− 1.682	*y* = 0.340*x* + 1.559
Q4	27	798 (630–1282)	254 (200–379)	*y* = 0.286*x* + 9.293	0.963	*y* = 0.244*x* − 29.053	*y* = 0.327*x* + 42.402
**Feline**	103	123 (75–224)	46 (27–79)	*y* = 0.358*x* + 0.432	0.997	*y* = 0.349*x* − 0.734	*y* = 0.368*x* + 1.328
**Human**	100	202 (175–241)	73 (62–84)	*y* = 0.337*x* + 2.959	0.944	*y* = 0.312*x* − 2.446	*y* = 0.363*x* + 8.121

JSCC: Japan Society of Clinical Chemistry.

IFCC: International Federation of Clinical Chemistry and Laboratory Medicine.

*r*: correlation coefficient.

### Regression between blood ALP measured by the JSCC and IFCC methods

**Fig 1:**
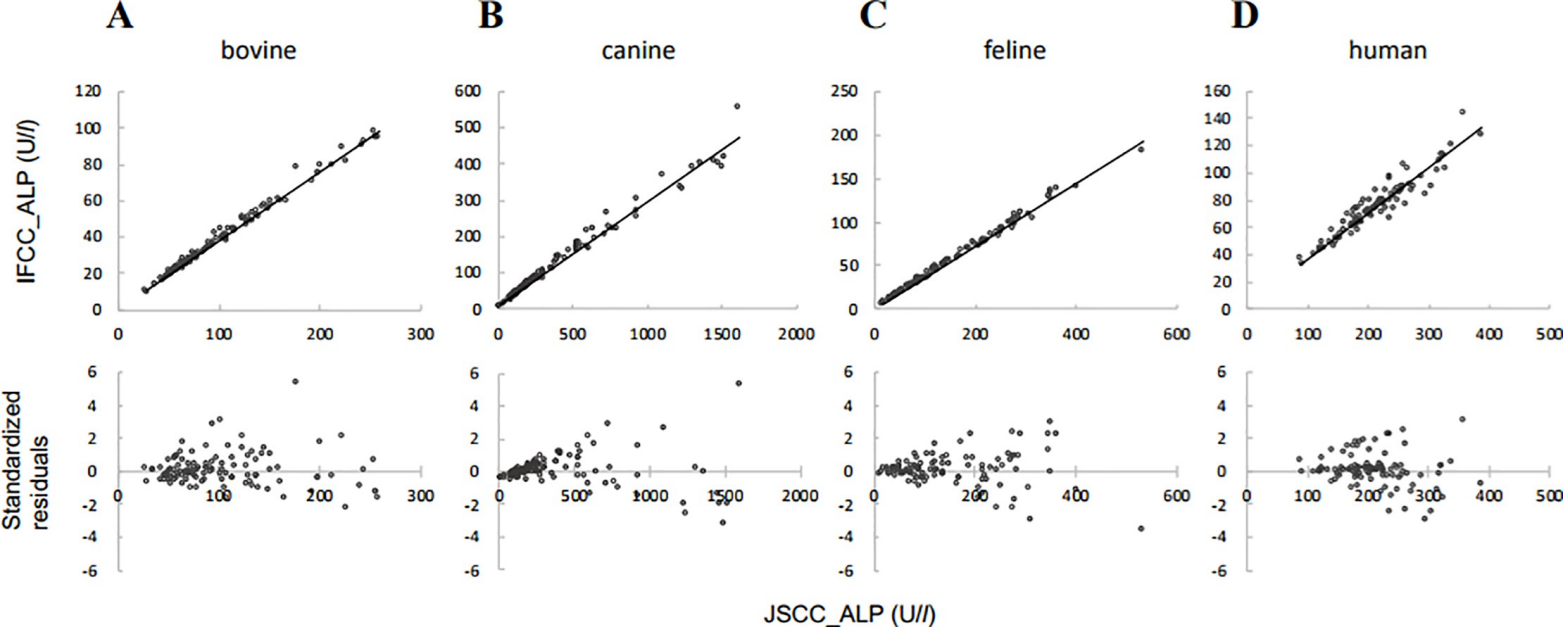
Scatter plots of the blood alkaline phosphatase (ALP) measured by Japan Society of Clinical Chemistry (JSCC) and International Federation of Clinical Chemistry and Laboratory Medicine (IFCC) reference methods and standardized residual plots from the regression formulas. Regression formulas are as follows: (A) 116 bovine specimens, *y* = 0.379*x* + 0.124, (B) 108 canine specimens, *y* = 0.289*x* + 8.291, (C) 103 feline specimens, *y* = 0.358*x* + 0.432, (D) 100 human specimens, *y* = 0.337*x* + 2.959.

Scatter plots of ALP values (Fig 1) produced well-behaved linear regressions. Correlation coefficients ranged from 0.944 to 0.997, and slope factors ranged from 0.289 to 0.380. Table 2 provides the 95% confidence intervals. Notably, regression formulas were *y* = 0.380*x* + 0.031 and *y* = 0.374*x* + 0.652 for the 105 Holstein Friesian and 11 Japanese Black cattle samples, respectively.

**Fig 2:**
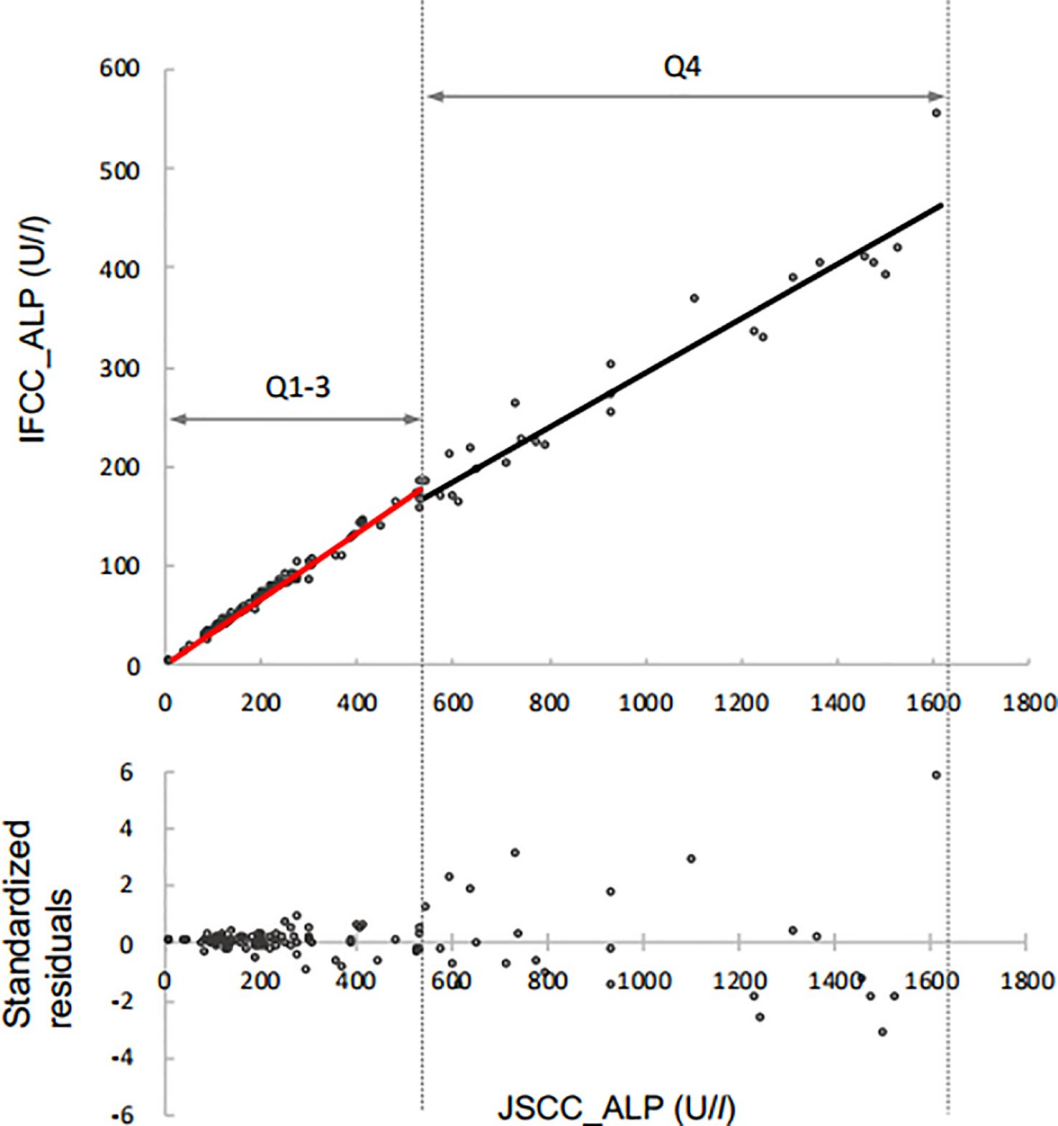
Results of the regression analysis between JSCC and IFCC in Q1–3 and Q4 in 108 canine samples, represented by the following equations: *y* = 0.331*x* − 0.045 and *y* = 0.286*x* + 9.293, respectively. Using these formulas, standardized residuals calculated in Q1–3 and Q4 showed no bias.

The standardized residual plots as per the regression equation are shown in Fig 1. Standardized residuals for each animal increased gradually with ALP concentration. The residual values were distributed equally in the standardized residual plots of bovine, feline, and human specimens. The fit of the regression line was not good because the variation in the low ALP concentration among canine specimens on the standardized residue plot was biased upwards (Fig 1). The canine specimens were divided into four groups after sorting the blood ALP activities in ascending order; quartile 1 (Q1), 15–142 U/L; Q2, 144–241 U/L; Q3, 253–532 U/L; and Q4, 535–1615 U/L. Regression equations were then examined by quartile. Regression formulas for Q1, Q2, Q3, and Q4 (lower and upper limit of 95% confidence intervals) were *y* = 0.336*x* − 0.450 (*y* = 0.324*x* − 2.086, *y* = 0.351*x* + 0.574), *y* = 0.368*x* − 6.501 (*y* = 0.338*x* − 14.621, *y* = 0.403*x* − 0.295), *y* = 0.347*x* − 7.127 (*y* = 0.322*x* − 19.076, *y* = 0.376*x* + 2.192), *y* = 0.286*x* + 9.293 (*y* = 0.244*x* − 29.053, *y* = 0.327*x* + 42.402), respectively. Standardized residual plots of Q1, Q2, Q3, and Q4 showed no bias (S1 Fig). Furthermore, the form of distribution of Q1, Q2, and Q3 residuals was similar (S1 Fig). Assessment of combined Q1, Q2, and Q3 data (Q1–3) produced a regression equation of *y* = 0.331*x* − 0.045 (*r* = 0.994) with an associated standardized residual plot without bias (Fig 2). The standardized residual plot of Q4 shows equal distribution, but greater variation is larger than the Q1–3 plot (Fig 2).

Next, we observed the systematic error. The value 1 was not included in the slope in the 95% confidence intervals for all the animal species, including humans. Therefore, they have a proportional systematic error. Essentially, proportional system error is natural as the measured value by the IFCC method differs widely from that of the JSCC method. In canine, the intercept does not contain the value “0” in the 95% confidence interval of the regression equations for all specimens. Therefore, there is a certain constant systematic error. In contrast, there is no constant systematic error in Specimen Q1-3 and Specimen Q4.

### Conversion factor between JSCC and IFCC methods measuring values

When the slope of the regression formula is used as the conversion coefficient, the conversion coefficient from the JSCC to the IFCC methods was as follows: bovine 0.379, canine (Q1-3) 0.331, canine (Q4) 0.286, feline 0.358, and human 0.337. The regression equation was calculated by the standard major axis regression method with the IFCC and JSCC measurement values as x and y, respectively. The conversion coefficients from the IFCC to JSCC methods for each animal species was as follows: bovine 2.639; canine (Q1-3) 3.025, (Q4) 3.500; feline 2.793; and human 2.968.

**Fig 3:**
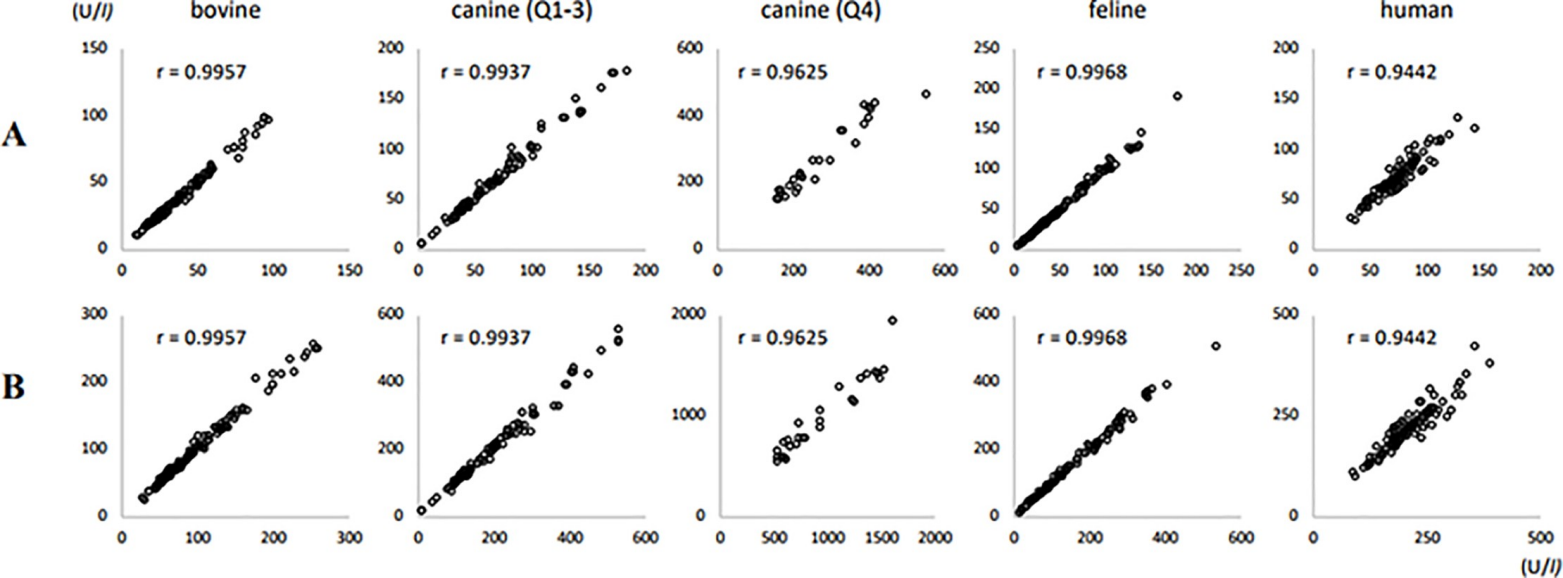
Scatter plots of the measured values (x-axis) and estimated values (y-axis) using the conversion factors. A: Measured IFCC values (x) and estimated IFCC values (y) obtained from the JSCC values using the conversion factors. B: Measured JSCC values (x) and estimated JSCC values (y) obtained from the IFCC values using the conversion factors.

Fig 3 shows scatter plots of the measured values (x) and estimated values (y) using the conversion factors. In bovine, canine (Q1-3), and feline, there was a high correlation between the estimated values and the measured values. In canine Q4 and humans, the correlation coefficient between estimates and measurements was lower than that in others.

## Discussion

This study indicated that bovine and feline blood ALP activities measured by the IFCC transferable method were roughly one-third of those determined by the JSCC transferable method, which was consistent with the findings using human and canine blood specimens. These results indicated that similar relationships might exist in other mammals. As described later, although there is some limitation, we showed the regressions and the provisional conversion formulas between the JSCC and IFCC methods in each of the animal species.

The regression formula comparing the JSCC (x) and IFCC methods (y) for randomly sampled human patient blood ALP was reported as *y* = 0.351*x* − 0.196 [6]. The regression formula of human specimens in our study, *y* = 0.337*x* + 2.959, is consistent with this report. In humans, the difference in ALP values between both methods occurs because of the difference in the composition of the reaction (buffer) solution used. Each human ALP isozyme activity measured by the IFCC method is lower than that analyzed using the JSCC method [2]. A similar change in enzymatic reactivity is likely to occur in the non-human mammal ALP isozymes.

The slope of the regression formulas is slightly different among animal species. Such differences could be influenced by variations in isozyme composition and activities among species. Blood ALP isozyme analysis using agarose gel electrophoresis indicated that liver-type ALP is predominant in adult bovines, canine, felines, and humans [5,7–9]. The next predominant ALP type among these animals is bone-type ALP. Although the concentration is lower than liver-type and bone-type ALPs, the intestine-type ALP is detected in bovine blood [8]. Felines and canines have intestinal ALP in blood that is generally undetectable because of its short half-life of a few minutes [9,10]. In canines, the cortisol-induced ALP, which is the same gene product as the intestine-type ALP, is present in the blood. Our previous study suggested that specimens with a high proportion of cortisol-induced ALP tended to deviate from the regression line and had activity like that of human intestinal ALP [5]. However, few studies on species differences in ALP isozyme activity are available. Therefore, the investigation of causes underlying differences in regression formulas is difficult.

The validity of ALP measurements in the present study was compared with the range of ALP values reported in previous studies. Regional differences in animal strains, diets, and breeding environments may affect ALP activity, and we compared our data with ALP data reported in Japan. The width of ALP distribution for Holstein Friesian cattle reported by Sato et al. [11] is similar to the width observed in the present study. No ALP measurements were found for mature Japanese black cattle, but animals under the age of one were more than four times the range of ALP in the present study [12]. This finding is likely due to an increase in bone-type ALP during rapid growth. Reference ranges of ALP in canines and felines reported in Japan are included within the ALP distribution in the present study with sufficient margin [13]. Therefore, the ALP values that we used for the regression analyses are appropriate for animal species assessed. The ALP range of the present study includes the Japanese ALP reference range for human blood samples [14].

We observed that standardized residuals increased gradually together with the ALP concentration (Fig 1). Activities of specific isozymes of ALP increase in response to bone growth, tissue damage, and bile stasis [1]. The relationship between JSCC and IFCC methods depends on the type of ALP isozyme. Therefore, regressions between the JSCC and IFCC methods are not constant at high ALP concentrations at which each specific isozyme concentration is increased.

The ALP distribution in canine blood measured in the present study was wider at high concentrations than in other animal species (Fig 1). The dog specimens represent residual blood from a clinical biological test requested by a veterinary hospital. Individual animals with a pathological condition with high ALP concentration were thus included. The slope of the regression line for canines was slightly different between the high concentrations and low and middle concentrations and likely reflects the wide distribution of canine ALP results. Canine regression equations developed separately for the low- and medium-concentration specimens (Q1–3) and the high-concentration samples (Q4) improved the distribution of the standardized residuals (Fig 2). We believed this approach enables more appropriate conversion between JSCC and IFCC method measurements for dogs. The slope of the regression formula for Q1–3 data for canines (*y* = 0.331*x* − 0.045) is consistent with the slopes of regression equations of other animals, including humans. Because the slope changes depending on the concentration range, we performed regression analysis using log-transformed values in a previous study [5]. The approach used this time when performing regression analysis without using logarithmic transformation values was also considered effective.

ALP isozymes, such as liver, bone, and intestine types, have different measured values even when the same analytical reagent is used, i.e., the reactivity of the isozyme is non-uniform even under the same measurement conditions. Furthermore, it is known that the reactivity changes non-uniformly when the buffer composition of the analytical reagent is different, as in JSCC and IFCC methods [2]. An example of a significant change in activity is intestinal ALP. Among all ALP isoenzymes, the small intestinal ALP has the highest reactivity with JSCC reagent. However, the intestinal ALP is the least responsive to the IFCC reagent compared with other isozymes. Even in other isozymes, there is no regularity in the change in activity associated with buffer changes. Because of this property, human specimens with a high proportion of specific isozymes, such as intestinal ALP and placental ALP, have been reported to deviate from the regression line between the JSCC and IFCC methods [4]. In humans with blood types B and O, the intestinal ALP in blood after food intake is higher than that in humans with blood types A and B. Therefore, specimens of blood types B and O often diverge from the regression line between the JSCC and IFCC methods [4]. In this study, the divergence from the regression line observed in the scatter plot for human samples is considered to be due to the presence of intestinal ALP depending on the blood type. As described above, the reactivity of human ALP isozyme with various ALP measurement reagents has been investigated. However, for animal ALP isozymes, changes in the activity of each measurement agent have not been sufficiently investigated.

In conclusion, when the ALP measurement values of the JSCC method were plotted on the x-axis and those of the IFCC method on the y-axis, the slope of the regression formula between both analytical methods was within 0.286–0.379 in the mature bovine, canine, feline, and human species. These regression formulas were similar, except for the high ALP region (Q4) in dogs. Probably, the regressions between the JSCC and IFCC ALP values in animals are affected by isozyme composition similar to what happens in humans. A bias toward a particular isozyme, such as an isozyme released during food intake, bone growth, pregnancy, or hepatobiliary system disease, may decrease the accuracy of estimated values using the conversion factors.

The present study has some limitations. First, the specimens used in this study do not cover the entire analytical range of ALP measurement reagents. In particular, the study in the high-concentration specimens is insufficient. Therefore, the regressions shown in this study could not be consistently maintained over the entire measurable range. Second, there might be sampling bias. Bovine, canine, and feline samples were collected from the residual blood from our medical analysis laboratory only. Similarly, human specimens were collected from only one laboratory. Thirdly, cryopreserved samples were used in this study. Freezing could have caused degradation or change in ALP activity. Finally, in this study, the reagent of only one company (Fujifilm Wako Pure Chemical) was examined. Although both reagents ALP II-J2 (JSCC method) and ALP IFCC (IFCC method) used in this study were standardized, the reactivity might not always be uniform with analytical reagents from other companies.

## Supporting information

S1 Fig Results of the regression analysis between JSCC and IFCC in Q1, Q2, Q3, and Q4 in 108 canine specimens, represented by the following equations: *y* = 0.336*x* − 0.450, *y* = 0.368*x* − 6.501, *y* = 0.347*x* − 7.127, and *y* = 0.286*x* + 9.293, respectively. Using these formulas, standardized residuals calculated in Q1–3 and Q4 showed no bias.
(TIFF)

S1 Dataset (XLSX)
