# Supplementary figures and images for: Comparison of regression for blood ALP levels using methods of the Japan Society of Clinical Chemistry and the International Federation of Clinical Chemistry and Laboratory Medicine in bovine, canine, feline, and human testing
Source: PLoS One. 2021 Jun 16;16(6):e0253396. doi: 10.1371/journal.pone.0253396 (PMC8208544; doi:10.1371/journal.pone.0253396)

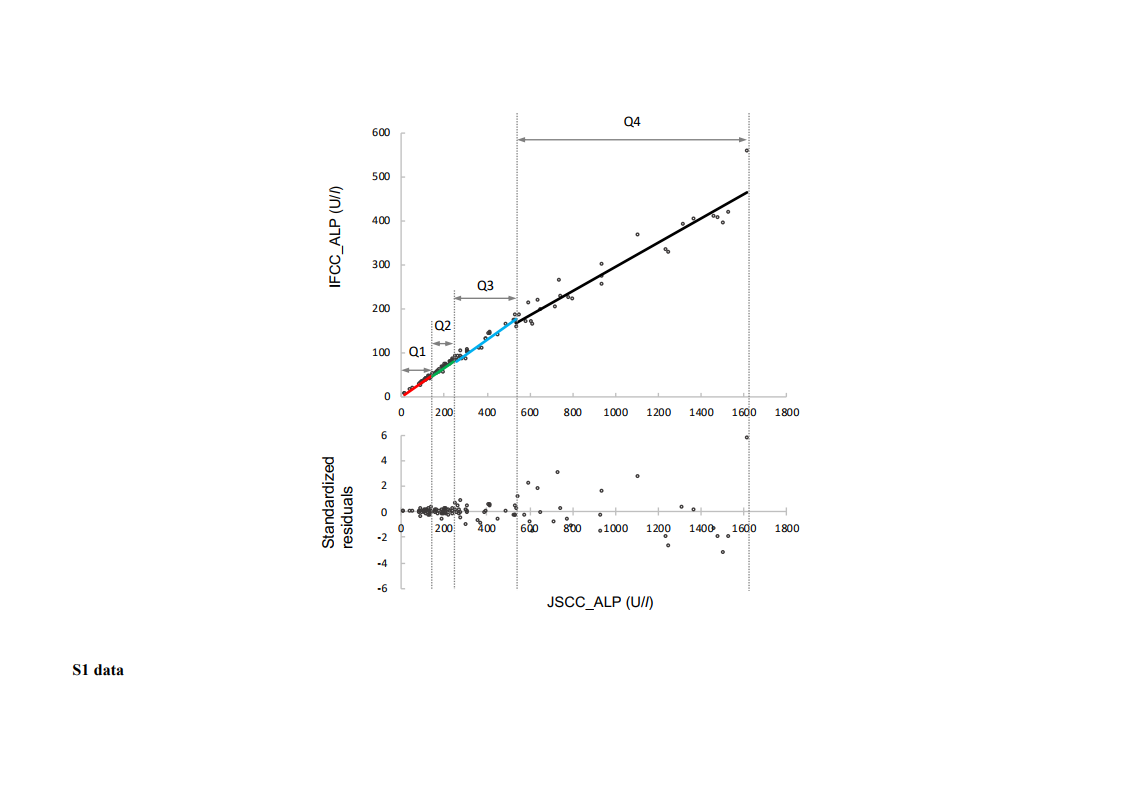

Supplement: S1 Fig — Using these formulas, standardized residuals calculated in Q1–3 and Q4 showed no bias. (TIFF) [file pone.0253396.s001.tiff]
